# Complications of patients with hematologic malignancies in a selected Iranian population

**DOI:** 10.22088/cjim.14.1.10

**Published:** 2023

**Authors:** Shadbahr Shayeghi, Mahtab Hamzeh, Ahmad Tamaddoni, Soraya Khafri, Farida Abesi

**Affiliations:** 1 Private Practice, Babol, Iran; 2 Non-Communicable Pediatric Diseases Research Center, Health Research Institute, Babol University of Medical Sciences, Babol, Iran; 3 Cancer Research Center, Health Research Institute, Babol University of Medical Sciences, Babol, Iran; 4 Dental Materials Research Center, Babol University of Medical Sciences, Babol, Iran

**Keywords:** Child, Hematologic malignancies, Radiography, Prevalence, Diagnosis

## Abstract

**Background:**

Hematologic malignancies in childhood and their treatments can cause dental anomalies and jaw bone abnormalities; therefore, the aim of this study was to assess the prevalence of these disorders in children.

**Methods:**

This cross-sectional study was conducted on all children diagnosed with hematologic malignancies that referred to in Amirkola Children’s Hospital from 2011 to 2018. All of children underwent clinical evaluation in a Dental Radiology Clinic and panoramic imaging was performed. The radiographs were examined for dental anomalies and jaw bone abnormalities. The data were analyzed by descriptive analysis using chi-square, Mann-Whitney and Kruskal-Wallis with a 95% confidence interval.

**Results:**

The study population consisted of 32 children and 9.46% of these patients showed intraoral signs in clinical evaluations. Panoramic radiographs indicated dental anomalies in 12 (63.9%) and jaw bone abnormalities in 17 (89.4%) patients. The most common dental anomaly and bone abnormality were taurodontism and changes in lamina dura, respectively. By measuring the mandibular cortical index (MCI), it was revealed that 13 had osteopenia, in which 4 of them had severe osteopenia (osteoporosis). The statistical analysis demonstrated that there was a significant difference between the incidence of osteoporosis and changes in lamina dura with the gender (p<0.05).

**Conclusion:**

Due to early onset of osteopenia and osteoporosis and the presence of dental and bone abnormalities in half of study population, early assessment of their clinical and radiographic condition can be helpful in the prevention and the early treatment of their oral complications.

Although the incidence rate of childhood cancer is estimated to be about 1 per 10000 children by World Health Organization (WHO), it varies among different populations because of predisposing factors like infections, genetic, and environmental factors (1, 2). Cancer is the third leading cause of death in Iran after cardiovascular diseases and car accidents (1). Leukemia, central nervous system tumors, and lymphoma are the most common cancers in childhood. According to the published studies, the incidence rate of childhood cancer in Iran is about 48-112 per million in females and 51-144 in males (3). Hematologic malignancies in childhood and its treatments can cause dental anomalies and jaw bone abnormalities.Lymphohematopoietic malignancies can have oral, dental and craniofacial manifestations in clinical and radiographic evaluation; for example, thrombocytopenia caused by leukemia can lead to Petechiae-like bleeding in the posterior region of the hard and soft palate or gingival bleeding (4). 

Malignant tumors can lead to resorption of alveolar bone, resulting in the floating-in-air appearance of teeth. Fast-growing malignant tumors spread through the easiest ways like maxillary sinus and ligament space, which can cause irregular widening and destruction of lamina dura (5). Chemotherapy, radiotherapy and stem cell transplantation are treatment modalities for hematopoietic childhood cancers. As dental and craniofacial development occurs in this age, these treatments can have adverse effects on odontogenesis (6). Radiotherapy can cause short root anomalies, hypodontia and changes in dental eruption. Chemotherapy can cause microdontia, hypodontia, taurodontism, shortening of roots and dental missing (7-9).

Dental anomalies have negative effects on the quality of life of childhood survivors. Survivors with enamel hypoplasia are more susceptible to caries formation because of colonization of oral bacteria. Although this fact displays the importance of annual dental examinations in these children; some studies have demonstrated that about 28% of childhood cancer survivors never receive any kind of dental services (10, 11). As mentioned above, the early diagnosis and preventive cares in children with hematopoietic malignancies are very important in the dental treatment prognosis and quality of life of these patients. Therefore, the aim of this study was to assess the prevalence of dental anomalies and jaw bone abnormalities in children's panoramic radiography.

## Methods

This cross-sectional study was conducted on all children diagnosed with lymphohematopoietic malignancies that referred to Amirkola Children’s Hospital from 2011 to 2018 and was approved by the Ethics Committee of Babol University of Medical Sciences with the code of IR.MUBABOL.REC.1397.021. All parents were informed of the study procedure. All patients were examined by a dentist and their clinical symptoms including gingival bleeding, petechiae, ecchymosis, gingival enlargement, mucositis, ulcer and swelling were recorded in a checklist and then, the patients were referred to an oral radiologist to perform panoramic imaging. The criteria for prescribing panoramic radiography was to have at least one decayed tooth in each quadrant.The radiographs were taken by a Cranex D (Soredex, Finland) with the following exposure attributes: KV = (57-85), mA = 10 mA, t = 11ms. The radiographs were observed by an oral radiologist and a pediatric dentist in a room with gleamy and indirect light on an LCD Flatron LG E1941 19-inch monitor (LG Electronics, Seoul, Korea). All children treated for at least six months were included in the study. The children with congenital dental anomalies and systemic diseases like diabetes and renal diseases were excluded.

The radiographs were analyzed for dental anomalies and bone abnormalities. The dental anomalies included microdontia, taurodontism, dental agenesis, root stunting and early closure of the apex. Jaw bone abnormalities composed of partial or complete loss of lamina dura, thickness of lamina dura, disappearance of the cortical border of the mandibular canal, disappearance of the cortical border of the maxillary sinus, periosteal reaction, transposition of the dental crypt and generalized loss of periodontal ligament. Mandibular cortical width (MCW) was measured on both sides, below the mental foramen, parallel to the long axis of the mandible and tangential to the inferior border; a line was drawn on the image using the Scanora software. Perpendicular to this tangent intersecting the inferior border of the mental foramen, a line was constructed along which the upper and lower delimitation points of the inferior mandibular cortex were located. Then, the MCW was measured.^ [^^12^^]^ Mandibular cortical index (MCI) is a way to evaluate osteopenia and osteoporosis in the cortical area of the mandible using panoramic radiographs. In this technique, the inferior cortex on both sides of the mandible, distal to the mental foramen is classified into three groups according to the following criteria:

1. C1: The endosteal margin of the cortex is even and sharp on both sides of the mandible, which is considered normal bone status.

2. C2: The endosteal margin has resorptive cavities with one to three layers of cortical residues on one or both sides, which is considered osteopenia.

3. C3: The endosteal margin consists of thick cortical residues and is clearly porous, which is considered severe osteopenia or osteoporosis.^ [^^13^^, ^^14^^]^

Data were analyzed by SPSS 25 using chi-square, Kruskal-Wallis and Mann-Whitney statistical tests. A p-value <0.05 was considered statistically significant.

## Results

The study population consisted of 16 males and 16 females with a mean age of 7.7 years (ranged 3.5-18). Twenty-nine patients were diagnosed with acute lymphoblastic leukemia (ALL), two cases with lymphoma and one with Langerhans cell histiocytosis. Twenty-eight patients were under treatment, and the therapy procedure of four of them was finished. Twenty-eight patients were treated by chemotherapy and four patients by both chemotherapy and radiotherapy. In clinical evaluation, 15 (46.9%) cases had intraoral signs including gingival enlargement (8 cases), oral ulcer (4 cases), mucositis (2 cases) and gingival bleeding (1 case). Only 19 of 32 participants underwent panoramic radiography. The analysis of these panoramic radiographs showed dental anomalies in 12, and bone developmental abnormalities in 17 patients. The most common dental anomaly was taurodontism (9 cases), followed by root stunting (6 cases), microdontia (2 cases), agenesis (2 cases) and abnormal root development (1 case).

Changes in lamina dura were the most common bone abnormality that occurred in 15 patients. Complete loss, partial loss and thickening of lamina dura were present in 7, 5, and 3 of patients, respectively. The disappearance of the mandibular canal border was present in 6 patients. Two patients developed generalized loss of periodontal ligament, and only one patient suggested disappearance of the cortical border of the maxillary sinus. Transposition of the dental crypt was present in one patient. There was no significant association between bone abnormalities, clinical examination, treatment type, and dental anomalies (p>0.05). There was a significant difference between men and women regarding MCI types. (Table 1)

**Table 1:** Correlation between MCI types and gender, clinical symptoms and treatment type in panoramic radiography

		**C1** **N (%)**	**C2** **N (%)**	**C3** **N (%)**	**P-value**
Gender	Male	5 (83.3)	2 (22.2)	3 (75)	0.041
	Female	1 (16.7)	7 (77.8)	1 (25)	
Clinical Symptoms	Ulcer	0 (0)	1 (11.1)	1 (25)	0.096
	Mucositis	2 (33.3)	0 (0)	0 (0)	
	Gingival enlargement	0 (0)	4 (44.4)	0 (0)	
	Normal	4 (66.7)	4 (44.4)	3 (75)	
Treatment Type	Chemotherapy	5 (83.3)	8 (88.8)	4 (100)	0.700
	Chemotherapy and Radiotherapy	1 (16.7)	1 (11.2)	0 (0)	

**Figure 1:**
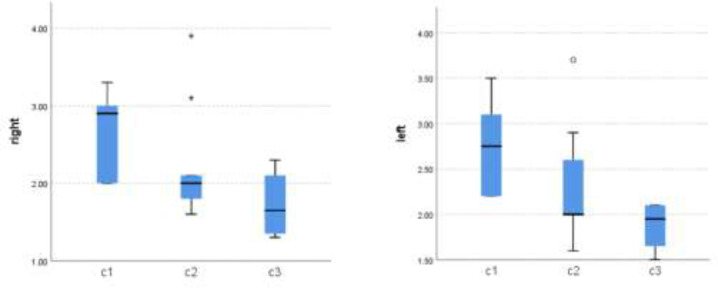
Comparison of mandibular cortical border thickness in different types of MCI between left and right

Analysis of MCI illustrated that C2 category was the most common type (9 cases). Four patients were categorized as C3, and six were classified as normal (C1). The mean of mandibular cortical border thickness in the right and left sides was 2.26 and 2.37, respectively. There was a significant association between the left cortical border thickness and C3 category (P=0.046) (table 2) (Figure 1). 

## Discussion

Most of the participants of the current study were children with ALL, who underwent chemotherapy. Chemotherapy in children may have oral manifestations such as mouth ulcers, gingival enlargement, gingival bleeding, petechia and ecchymosis (15, 16). The majority of the subjects of the present study had oral complications including mouth ulcers, gingival enlargement, mucositis and gingival bleeding. Gingival enlargement was the most common oral manifestation.

Velten et al. conducted a study in Brazil on the oral complications of chemotherapy and found that mucositis was the most common oral complication (17). Ponce-Torres et al. performed another study on oral complications of children with ALL who underwent chemotherapy in Mexico. They concluded that gingivitis was the most common oral complication (18). Differences in the frequency of oral lesions in various studies can be due to the differences in the study populations, used medications and the criteria of the clinical trials. Since it was impossible to use a periodontal probe because of the low age and uncooperativeness of the patients and their unfavorable condition, it was not feasible to measure the depth of the pocket, and thereby examine the periodontal condition and categorize the gingivitis and periodontitis. In addition, mucositis is an early complication of chemotherapy, and in most cases, it was not detectable on the examination day. So, the percentage of patients with mucositis cannot be precise. Out of 32 participants, only 19 subjects underwent panoramic radiography. Although panoramic radiography and its subsequent treatments were provided free of charge, some of the parents refused to cooperate, indicating the lack of awareness regarding the impact of oral health on the quality of life of these children. In the ongoing study, 63.1% of the patients had dental anomalies such as taurodontism and root resorption, which were the most common dental anomalies among them. According to the results of a study conducted by Minicucci et al. on children with ALL who underwent chemotherapy in Brazil, 82.9% of them had dental anomalies including microdontia and root resorption which were the most common abnormalities based on the obtained radiographs (6). This result is inconsistent with that of the present study since only two cases of microdontia were observed in the current study. This inconsistency could be due to the fact that microdontia can be genetic and not related to the treatment of malignant diseases (5). Moreover, tooth formation defects are the late complication of chemotherapy and radiotherapy so maybe some cases of tooth formation defects were not detected in panoramic radiography at the time of examination.

Khojastehpour et al. in Iran (2014) evaluated the prevalence of anomalies and dental age in 25 children with ALL who underwent chemotherapy. The results indicated that chemotherapy led to dental anomalies, but did not affect the dental age, puberty or tooth development.^ [^^8^^]^ Similarly, in the present study, only one case experienced the premature closure of the developing tooth apex. According to the study performed by Gawade et al., (19) van der Pas-van Voskuilen et al (20). and Vesterbacka et al (21). bone marrow transplantation has long-term negative effects on children's dental development and can lead to developmental dental disorders such as agenesis, root resorption and cessation of root development. In the present study, none of the children underwent bone marrow transplantation.

According to the results of a systematic study carried out by Silva et al., radiographs of patients with lymphoma indicated an increase in the thickness of the periodontal ligament and loss of lamina dura. The loss of lamina dura in these patients is usually due to the infiltration of malignant cells; moreover, it can be owing to the release of osteoclast-activating factors from lymphoid cells (22). In the ongoing study, bone disorders were observed in about 90% of patients, most of which were changes in lamina dura. In another study performed by Sugihara et al. on 10 children with ALL and AML, the loss of lamina dura and disappearance of the inferior alveolar canal border were seen in five and seven cases, respectively (23).

In the current study, the status of osteopenia was examined according to the MCI. To do so, the radiographs were divided into three categories of C1, C2, and C3 based on MCI and according to the appearance of the inferior border of the mandible (13). According to this index, out of 19 participants, nine and four cases had osteopenia and osteoporosis, respectively. Furthermore, there was a significant relationship between C3 type and thickness of the left cortical border of the mandible. According to the findings of a study performed by Wasilewski-Masker et al., metabolic changes caused by childhood cancer treatment can interfere with the attainment of peak bone mass and lead to premature onset of osteopenia, osteoporosis and more serious problems (24).

Based on the results, there was an early onset of osteopenia and osteoporosis in children treated with anti-cancer therapies. Since bone mineral deficiency after treatment of various types of childhood cancers can be accompanied by an increased risk of bone fractures and other problems caused by osteopenia and osteoporosis, early detection and lifestyle changes can prevent them or reduce their severity.

In conclusion due to early onset of osteopenia and osteoporosis and presence of dental and bone abnormalities in half of study population, early assessment of their clinical and radiographic condition can be helpful in the prevention and the early treatment of their oral complications
